# Bis[tris­(ethyl­enediamine-κ^2^ *N*,*N*′)cobalt(III)] octa­kis-μ-_3_-oxido-hexa­deca-μ_2_-oxido-tetra­deca­oxido-μ_12_-tetra­oxo­silicato-octa­molybdenum(VI)hexa­vanadium(IV,V) hexa­hydrate

**DOI:** 10.1107/S1600536811048197

**Published:** 2011-11-19

**Authors:** Yu-Kun Lu, Ming-Ming Tian, Shu-Gang Xu, Ren-Qing Lü, Yun-Qi Liu

**Affiliations:** a College of Science and State Key Laboratory of Heavy Oil Processing, China University of Petroleum (East China), Qingdao Shandong 266555, People’s Republic of China; b College of Chemical Engineering and State Key Laboratory of Heavy Oil Processing, China University of Petroleum (East China), Qingdao Shandong 266555, People’s Republic of China

## Abstract

The title compound, [Co(C_2_H_8_N_2_)_3_]_2_[SiMo_8_V_4_O_40_(VO)_2_]·6H_2_O, was prepared under hydro­thermal conditions. The asymmetric unit consists of a transition metal complex [Co(en)_3_]^3+^ cation (en is ethyl­enediamine), one half of an [SiMo_8_V_4_O_40_(VO)_2_]^6−^ heteropolyanion, two solvent water mol­ecules in general positions and two half-mol­ecules of water located on a mirror plane. In the complex cation, the Co^3+^ ion is in a distorted octa­hedral coordination environment formed by six N atoms of the three chelating en ligands. One of the en ligands exhibits disorder of its aliphatic chain over two sets of sites of equal occupancy. The [SiMo_8_V_4_O_40_(VO)_2_]^6−^ heteropolyanion is a four-electron reduced bivanadyl-capped α-Keggin-type molybdenum–vanadium–oxide cluster. In the crystal, it is located on a mirror plane, which results in disorder of the central tetra­hedral SiO_4_ group: the O atoms of this group occupy two sets of sites related by a mirror plane. Furthermore, all of the eight μ_2_-oxide groups are also disordered over two sets of sites with equal occupancy. There are extensive inter­molecular N—H⋯O hydrogen bonds between the complex cations and inorganic polyoxidoanions, leading to a three-dimensional supra­molecular network.

## Related literature

For general background to polyoxidometalates, see: Pope & Müller (1991 ▶); Hill (1998 ▶); Kurth *et al.* (2001 ▶). For bicapped Keggin-type anions, see: Chen & Hill (1996 ▶); Lu, Cui, Liu *et al.* (2009 ▶); Lu, Cui *et al.* (2010 ▶); Lu, Xu & Yu (2010 ▶); Luan *et al.* (2002 ▶); Müller *et al.* (1994 ▶); Xu *et al.* (1998 ▶). For general background to bond-valence calculations, see: Brown & Altermatt (1985 ▶). For the structure and chemistry of reduced heteropolyanions, see: Khan *et al.* (1993 ▶); Lu, Cui, Chen *et al.* (2009 ▶), Lu, Xu, Cui *et al.* (2010 ▶); Müller *et al.* (1994 ▶).
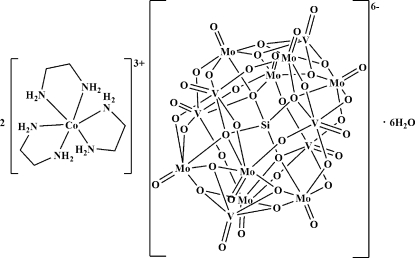


## Experimental

### 

#### Crystal data

[Co(C_2_H_8_N_2_)_3_]_2_[SiMo_8_V_4_O_40_(VO)_2_]·6H_2_O
*M* *_r_* = 2359.83
Orthorhombic, 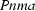

*a* = 20.744 (4) Å
*b* = 21.498 (4) Å
*c* = 13.623 (3) Å
*V* = 6075 (2) Å^3^
*Z* = 4
Mo *K*α radiation
μ = 3.13 mm^−1^
*T* = 293 K
0.22 × 0.21 × 0.19 mm

#### Data collection

Rigaku R-AXIS RAPID diffractometer
Absorption correction: multi-scan (*ABSCOR*; Higashi, 1995 ▶) *T*_min_ = 0.508, *T*_max_ = 0.552
45336 measured reflections
5404 independent reflections
3951 reflections with *I* > 2σ(*I*)
*R*_int_ = 0.080

#### Refinement

*R*[*F* ^2^ > 2σ(*F* ^2^)] = 0.054
*wR*(*F* ^2^) = 0.133
*S* = 1.05
5404 reflections
505 parameters
H-atom parameters constrained
Δρ_max_ = 2.04 e Å^−3^
Δρ_min_ = −1.07 e Å^−3^

### 

Data collection: *PROCESS-AUTO* (Rigaku, 1998 ▶); cell refinement: *PROCESS-AUTO*; data reduction: *PROCESS-AUTO*; program(s) used to solve structure: *SHELXS97* (Sheldrick, 2008 ▶); program(s) used to refine structure: *SHELXL97* (Sheldrick, 2008 ▶); molecular graphics: *DIAMOND* (Brandenburg, 1999 ▶); software used to prepare material for publication: *SHELXL97*.

## Supplementary Material

Crystal structure: contains datablock(s) I, global. DOI: 10.1107/S1600536811048197/gk2413sup1.cif

Structure factors: contains datablock(s) I. DOI: 10.1107/S1600536811048197/gk2413Isup2.hkl

Additional supplementary materials:  crystallographic information; 3D view; checkCIF report

## Figures and Tables

**Table 1:** Hydrogen-bond geometry (Å, °)

*D*—H⋯*A*	*D*—H	H⋯*A*	*D*⋯*A*	*D*—H⋯*A*
N1—H1*C*⋯O4^i^	0.90	2.08	2.918 (10)	154
N1—H1*D*⋯O14^ii^	0.90	2.48	2.988 (16)	117
N2—H2*D*⋯O10^iii^	0.90	2.24	3.040 (9)	148
N2—H2*C*⋯O12^iv^	0.90	2.22	3.034 (11)	151
N3—H3*D*⋯O19^ii^	0.90	1.97	2.765 (15)	147
N4—H4*D*⋯O3^v^	0.90	2.17	3.042 (9)	164
N4—H4*C*⋯O15^vi^	0.90	2.09	2.770 (16)	131
N5—H5*D*⋯O7^ii^	0.90	2.26	2.923 (10)	130
N5—H5*C*⋯O7^vi^	0.90	2.12	2.905 (11)	145
N5—H5*D*⋯O13^ii^	0.90	2.07	2.835 (18)	142
N6—H6*C*⋯O4^i^	0.90	2.31	3.126 (11)	150
N6—H6*D*⋯O10^iii^	0.90	2.57	3.109 (9)	120
N6—H6*C*⋯O18^i^	0.90	2.37	2.972 (11)	124

Symmetry codes: (i) 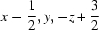; (ii) 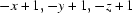; (iii) 
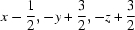
; (iv) 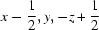; (v) 
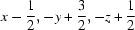
; (vi) 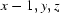.

**Fig. 1.**
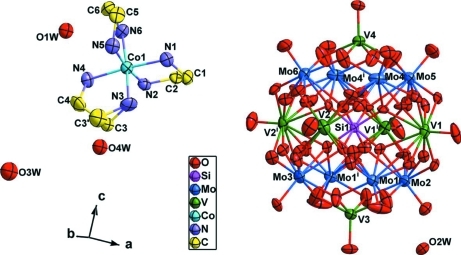
A view of the molecule of title compound with displacement ellipsoids drawn at the 50% probability level. H atoms have been omitted. [Symmetry codes: (i) x, 1.5 - y, z]

**Fig. 2.**
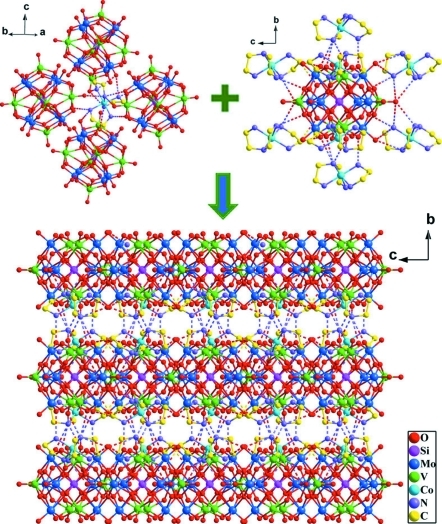
Ball-and-stick representation of the three-dimensional supramolecular network structure of the title compound. Dashed lines represent hydrogen bonds.

## supplementary crystallographic information

### Comment

The design and synthesis of polyoxometalates (POMs) has attracted continuous research interest not only because of their appealing structural and topological novelties, but also due to their interesting optical, electronic, magnetic, and catalytic properties, as well as their potential medical applications (Pope & Müller, 1991; Hill, 1998). Most of the structures in these compounds contain fragments of several well known polyoxoanions, such as Keggin, Lindquist and Dawson anion, or their derivatives, which are the basis for numerous POMs. The Keggin-type structure was of epoch-making signifcance in the history of POM chemistry (Kurth, 2001). In our research group, transition metal complexes (TMCs), such as [Ni(phen)_3_]^2+^, [Ni(en)_3_]^2+^, [Ni(en)_2_(H_2_O)_2_]^2+^, [Ni(en)_2_]^2+^ and [Cu(en)_2_(H_2_O)]^2+^, are used to effectively modify POMs under hydrothermal conditions (Lu, Cui, Liu *et al.* 2009; Lu, Cui, Chen *et al.*. 2009). Here, we describe the synthesis and structural characterization of the title compound.

As shown in Fig. 1, single crystal X-ray diffraction analysis reveals that the title compound consists of a bi-capped α-Keggin polyoxoanion [SiMo_8_V_4_O_40_(VO)_2_]^6–^, two TMCs countercations [Co(en)_3_]^3+^ and six lattice water molecules. The Co^III^ ion is in a distorted octahedral coordination geometry, bonded by six N atoms from the three chelating en ligands. The Co—N bond lengths are in the range of 1.948 (8)–1.975 (8) Å. The heteropolyanion [SiMo_8_V_4_O_40_(VO)_2_]^6–^ can be described as a pseudo-Keggin core [SiMo_8_V_4_O_40_]^10–^ with two additional five-coordinate terminal {VO}^2+^ units capping two opposite {Mo_4_O_4_} square holes. This α-Keggin core [SiMo_8_V_4_O_40_]^10–^, similar to those of [XMo_8_V_4_O_40_]^n–^ (*X*= Si, As and P) (Luan *et al.*, 2002; Müller *et al.*, 1994; Xu *et al.*, 1998), consists of four internally edge-shared octahedral {Mo_2_VO_13_} connected with each other by corner-sharing oxygen atoms and enwrapping the central disordered SiO_4_ tetrahedron; to look at it another way, it also a sandwich structure consisting of two {Mo_4_} rings and one {V_4_} belt distributed alternately. Furthermore, all of the eight doubly bridging oxide groups (O13–O16, O19–O22) are also disordered with the occupancy factor of 0.5 for each O atom.

The SiO_4_ tetrahedron has Si—O distances of 1.623 (10)–1.674 (11) Å and bond angles in the range of 108.1 (5)–110.9 (6)°.V1, V2 and all Mo atoms have a distorted {MO_6_} octahedral environment, and the capped V atoms (V3 and V4) show a distorted {VO_5_} square pyramidal geometry, respectively. According to the kind of oxygen atoms bonded to the Mo/V atoms, the Mo/V–O bond lengths are divided in three groups: Mo—O_c_ 2.365 (11)–2.429 (11) Å, V—O_c_ 2.328 (11)–2.454 (10) Å (O_c_, central O atoms); Mo—O_b_ 1.643 (15)–2.067 (6) Å, V—O_b_ 1.794 (16)–2.156 (17) Å (O_b_, bridged O atoms); Mo—O_t_ 1.656 (10)–1.698 (8), V—O_t_ 1.573 (7)–1.649 (8) Å (O_t_, terminal O atoms). All of the Si—O, Mo—O and V—O bond lengths are within the normal ranges.

The bond valence sums (BVS) for the Mo and V centers were calculated by using parameters given by Brown (Brown & Altermatt, 1985). The values are 6.15, 6.21, 6.32, 6.30, 6.12 and 6.29 for Mo1, Mo2, Mo3, Mo4, Mo5 and Mo6, and the calculated valence sums for V1, V2, V3 and V4 are 4.47, 4.42, 4.16 and 3.96, respectively. The calculated results indicate that the oxidation state of all Mo centers are +6, V1 and V2 centers have a mixed valence state (+4 and +5), and the capped V atoms are +4, respectively; similar to the reported representative (Xu *et al.*, 1998). We consider that oxalic acid acts as reducing agent reducing V^V^ to V^IV^ in the synthesis.

The molecules are linked into a three-dimensional network by a combination of intermolecular N—H···O and C—H···O hydrogen bonds (Fig. 2).

### Experimental

A mixture of Na_2_SiO_3_.9H_2_O (0.28 g, 1 mmol), MoO_3_.2H_2_O (0.54 g, 3.0 mmol), V_2_O_5_ (0.54 g, 3.0 mmol), Co(NO_3_)_2_.6H_2_O (0.44 g, 1.5 mmol), C_2_H_2_O_4_.2H_2_O (0.25 g, 2.0 mmol) and 18 ml water was stirred for 2 h in air; it was adjusted to pH = 6 with en and was heated in a 30 ml stainless steel reactor with a Teflon-liner at 180°C for 6 days, and then cooled to room temperature. Black prism crystals were isolated with 55% yield (based on Mo). Elemental analysis: calcd: C, 6.11; H, 2.56; N, 7.12; found: C, 6.09; H, 2.51; N, 7.18.

### Refinement

H atoms bonded to C and N atoms were positioned geometrically and refined as riding atoms, with C–H = 0.97 Å, N–H = 0.90 Å and *U*_iso_(H) = 1.2Ueq(C, N). The hydrogen atoms of four crystallographic water molecules could not be located unambiguously from difference Fourier maps, probably due to disorder of the water molecules.Thus the structure was refined without the H atoms of the water molecules (which include the water O atoms O1W, O2W, O3W and O4W). In the SiO_4_ unit, the four oxygen atoms (O23—O26) are equally disordered about the mirror plane. All of eight µ2-oxide groups are also disordered with an occupancy factor of 0.5 for each O atom. In complex cation, the C3 and C3' atoms were disordered with a 0.5 occupancy and refined isotropically. In the final difference Fourier map, the highest residual electron density was found at 1.74 Å away from O1W atom and the deepest hole at 0.92 Å from Mo4.

### Crystal data

**Table:**

[Co(C_2_H_8_N_2_)_3_]_2_[Mo_8_V_6_O_42_Si]·6H_2_O	*F*(000) = 4568
*M**_r_* = 2359.83	*D*_x_ = 2.580 Mg m^−^^3^
Orthorhombic, *P**n**m**a*	Mo *K*α radiation, λ = 0.71073 Å
Hall symbol: -P 2ac 2n	Cell parameters from 4213 reflections
*a* = 20.744 (4) Å	θ = 2.2–24.6°
*b* = 21.498 (4) Å	µ = 3.13 mm^−^^1^
*c* = 13.623 (3) Å	*T* = 293 K
*V* = 6075 (2) Å^3^	Prism, black
*Z* = 4	0.22 × 0.21 × 0.19 mm

### Data collection

**Table:**

Rigaku R-AXIS RAPID diffractometer	5404 independent reflections
Radiation source: fine-focus sealed tube	3951 reflections with *I* > 2σ(*I*)
graphite	*R*_int_ = 0.080
Detector resolution: 10 pixels mm^-1^	θ_max_ = 25.0°, θ_min_ = 2.0°
ω scans	*h* = −24→24
Absorption correction: multi-scan (*ABSCOR*; Higashi, 1995)	*k* = −25→25
*T*_min_ = 0.508, *T*_max_ = 0.552	*l* = −16→16
45336 measured reflections	

### Refinement

**Table:**

Refinement on *F*^2^	Primary atom site location: structure-invariant direct methods
Least-squares matrix: full	Secondary atom site location: difference Fourier map
*R*[*F*^2^ > 2σ(*F*^2^)] = 0.054	Hydrogen site location: inferred from neighbouring sites
*wR*(*F*^2^) = 0.133	H-atom parameters constrained
*S* = 1.05	*w* = 1/[σ^2^(*F*_o_^2^) + (0.0462*P*)^2^ + 70.2923*P*] where *P* = (*F*_o_^2^ + 2*F*_c_^2^)/3
5404 reflections	(Δ/σ)_max_ = 0.001
505 parameters	Δρ_max_ = 2.04 e Å^−^^3^
0 restraints	Δρ_min_ = −1.07 e Å^−^^3^

### Special details

**Table:**

Geometry. All e.s.d.'s (except the e.s.d. in the dihedral angle between two l.s. planes) are estimated using the full covariance matrix. The cell e.s.d.'s are taken into account individually in the estimation of e.s.d.'s in distances, angles and torsion angles; correlations between e.s.d.'s in cell parameters are only used when they are defined by crystal symmetry. An approximate (isotropic) treatment of cell e.s.d.'s is used for estimating e.s.d.'s involving l.s. planes.
Refinement. Refinement of *F*^2^ against ALL reflections. The weighted *R*-factor *wR* and goodness of fit *S* are based on *F*^2^, conventional *R*-factors *R* are based on *F*, with *F* set to zero for negative *F*^2^. The threshold expression of *F*^2^ > σ(*F*^2^) is used only for calculating *R*-factors(gt) *etc*. and is not relevant to the choice of reflections for refinement. *R*-factors based on *F*^2^ are statistically about twice as large as those based on *F*, and *R*- factors based on ALL data will be even larger.

### Fractional atomic coordinates and isotropic or equivalent isotropic displacement parameters (Å^2^)

**Table:**

	*x*	*y*	*z*	*U*_iso_*/*U*_eq_	Occ. (<1)
Si1	0.79072 (15)	0.7500	0.4979 (2)	0.0178 (6)	
Mo1	0.81179 (4)	0.62748 (3)	0.32716 (5)	0.0268 (2)	
Mo2	0.93678 (6)	0.7500	0.36362 (9)	0.0397 (3)	
Mo3	0.68820 (6)	0.7500	0.28998 (9)	0.0421 (3)	
Mo4	0.76924 (5)	0.62768 (4)	0.66713 (6)	0.0417 (3)	
Mo5	0.89334 (6)	0.7500	0.70708 (8)	0.0315 (3)	
Mo6	0.64507 (5)	0.7500	0.63260 (9)	0.0307 (3)	
V1	0.90955 (7)	0.63391 (7)	0.53319 (12)	0.0301 (4)	
V2	0.67036 (8)	0.63275 (7)	0.46240 (13)	0.0362 (4)	
V3	0.82853 (10)	0.7500	0.20876 (15)	0.0262 (5)	
V4	0.75451 (10)	0.7500	0.78757 (15)	0.0263 (5)	
Co1	0.15902 (6)	0.58507 (5)	0.49030 (9)	0.0294 (3)	
O1	0.8208 (5)	0.5771 (3)	0.2354 (5)	0.069 (3)	
O2	1.0004 (4)	0.7500	0.2891 (7)	0.041 (2)	
O3	0.6514 (4)	0.7500	0.1786 (6)	0.032 (2)	
O4	0.7561 (3)	0.5756 (3)	0.7579 (5)	0.0425 (17)	
O5	0.9315 (5)	0.7500	0.8150 (8)	0.066 (3)	
O6	0.5836 (5)	0.7500	0.7100 (8)	0.068 (4)	
O7	0.9608 (4)	0.5803 (3)	0.5471 (6)	0.059 (2)	
O8	0.6172 (3)	0.5813 (3)	0.4485 (6)	0.055 (2)	
O9	0.8449 (5)	0.7500	0.0929 (6)	0.042 (2)	
O10	0.7337 (4)	0.7500	0.9044 (6)	0.032 (2)	
O11	0.8795 (3)	0.6921 (3)	0.2825 (6)	0.051 (2)	
O12	0.7616 (3)	0.6916 (3)	0.2466 (6)	0.0496 (19)	
O13	0.8744 (8)	0.5921 (7)	0.4047 (12)	0.024 (4)	0.50
O13'	0.8578 (8)	0.6084 (8)	0.4344 (12)	0.028 (4)	0.50
O14	0.7342 (7)	0.5892 (7)	0.3626 (11)	0.028 (3)	0.50
O14'	0.7450 (7)	0.6127 (7)	0.4009 (12)	0.033 (4)	0.50
O15	0.9674 (7)	0.6750 (7)	0.4339 (12)	0.031 (4)	0.50
O15'	0.9469 (7)	0.6987 (7)	0.4560 (11)	0.025 (3)	0.50
O16	0.6450 (7)	0.6751 (6)	0.3373 (11)	0.030 (3)	0.50
O16'	0.6558 (7)	0.7021 (7)	0.3700 (11)	0.031 (3)	0.50
O17	0.8201 (3)	0.6912 (3)	0.7494 (6)	0.051 (2)	
O18	0.7034 (4)	0.6918 (3)	0.7116 (6)	0.050 (2)	
O19	0.8543 (6)	0.5898 (6)	0.6344 (10)	0.025 (3)	0.50
O19'	0.8298 (6)	0.6092 (6)	0.5944 (9)	0.024 (3)	0.50
O20	0.7004 (8)	0.5936 (6)	0.5894 (10)	0.029 (3)	0.50
O20'	0.7329 (7)	0.6084 (7)	0.5620 (11)	0.037 (4)	0.50
O21	0.9410 (8)	0.6804 (7)	0.6581 (11)	0.026 (3)	0.50
O21'	0.9223 (8)	0.6960 (7)	0.6245 (12)	0.030 (4)	0.50
O22	0.6116 (8)	0.6835 (9)	0.5654 (14)	0.036 (4)	0.50
O22'	0.6377 (8)	0.6928 (8)	0.5391 (13)	0.030 (4)	0.50
O23	0.8434 (5)	0.7057 (5)	0.4442 (8)	0.024 (3)	0.50
O24	0.7554 (5)	0.7053 (5)	0.4148 (8)	0.022 (2)	0.50
O25	0.7361 (5)	0.7084 (5)	0.5529 (8)	0.025 (3)	0.50
O26	0.8291 (5)	0.7039 (5)	0.5777 (8)	0.025 (3)	0.50
O1W	0.0645 (5)	0.7500	0.5774 (7)	0.044 (2)	
O2W	0.9546 (8)	0.6028 (10)	0.1408 (12)	0.185 (7)	
O3W	1.007 (2)	0.7500	0.066 (3)	0.288 (18)*	
O4W	0.0681 (10)	0.4140 (10)	0.1905 (15)	0.206 (8)*	
N1	0.2251 (4)	0.5308 (4)	0.5456 (6)	0.0379 (19)	
H1C	0.2258	0.5350	0.6113	0.045*	
H1D	0.2156	0.4909	0.5315	0.045*	
N2	0.2313 (4)	0.6400 (3)	0.4554 (6)	0.0338 (18)	
H2C	0.2358	0.6411	0.3897	0.041*	
H2D	0.2230	0.6789	0.4764	0.041*	
N3	0.1632 (5)	0.5377 (4)	0.3662 (6)	0.048 (2)	
H3C	0.2047	0.5287	0.3533	0.058*	
H3D	0.1422	0.5014	0.3743	0.058*	
N4	0.0946 (4)	0.6364 (3)	0.4228 (6)	0.040 (2)	
H4C	0.0613	0.6441	0.4632	0.048*	
H4D	0.1123	0.6731	0.4053	0.048*	
N5	0.0914 (4)	0.5316 (3)	0.5451 (6)	0.039 (2)	
H5C	0.0530	0.5416	0.5187	0.047*	
H5D	0.0998	0.4915	0.5308	0.047*	
N6	0.1472 (4)	0.6323 (4)	0.6110 (6)	0.0370 (19)	
H6C	0.1838	0.6307	0.6464	0.044*	
H6D	0.1398	0.6724	0.5958	0.044*	
C1	0.2897 (5)	0.5464 (5)	0.5050 (8)	0.043 (2)	
H1A	0.2949	0.5289	0.4399	0.051*	
H1B	0.3236	0.5303	0.5471	0.051*	
C2	0.2923 (4)	0.6171 (4)	0.5011 (7)	0.037 (2)	
H2A	0.2967	0.6339	0.5669	0.044*	
H2B	0.3291	0.6305	0.4626	0.044*	
C3	0.1369 (11)	0.5689 (10)	0.2849 (15)	0.038 (5)*	0.50
H3A	0.1255	0.5399	0.2330	0.046*	0.50
H3B	0.1666	0.5997	0.2592	0.046*	0.50
C3'	0.1067 (15)	0.5577 (14)	0.298 (2)	0.072 (8)*	0.50
H3'1	0.1243	0.5706	0.2357	0.086*	0.50
H3'2	0.0794	0.5219	0.2866	0.086*	0.50
C4	0.0713 (6)	0.6024 (6)	0.3326 (8)	0.060 (3)	
H4A	0.0522	0.6312	0.2861	0.072*	
H4B	0.0394	0.5713	0.3501	0.072*	
C5	0.0891 (5)	0.5400 (5)	0.6519 (8)	0.047 (3)	
H5A	0.1251	0.5187	0.6827	0.057*	
H5B	0.0494	0.5229	0.6781	0.057*	
C6	0.0927 (5)	0.6084 (5)	0.6722 (8)	0.041 (2)	
H6A	0.0526	0.6286	0.6540	0.049*	
H6B	0.1008	0.6159	0.7413	0.049*	

### Atomic displacement parameters (Å^2^)

**Table:**

	*U*^11^	*U*^22^	*U*^33^	*U*^12^	*U*^13^	*U*^23^
Si1	0.0198 (15)	0.0138 (14)	0.0199 (16)	0.000	−0.0007 (13)	0.000
Mo1	0.0369 (4)	0.0179 (4)	0.0257 (4)	0.0019 (3)	−0.0021 (3)	−0.0007 (3)
Mo2	0.0262 (6)	0.0633 (9)	0.0295 (7)	0.000	0.0013 (5)	0.000
Mo3	0.0292 (6)	0.0651 (9)	0.0321 (7)	0.000	−0.0075 (5)	0.000
Mo4	0.0806 (7)	0.0171 (4)	0.0273 (4)	−0.0061 (4)	0.0081 (4)	−0.0004 (3)
Mo5	0.0343 (6)	0.0316 (6)	0.0287 (6)	0.000	−0.0043 (5)	0.000
Mo6	0.0295 (6)	0.0271 (6)	0.0354 (7)	0.000	0.0054 (5)	0.000
V1	0.0286 (8)	0.0191 (7)	0.0427 (9)	0.0037 (6)	0.0029 (7)	0.0059 (7)
V2	0.0369 (9)	0.0250 (8)	0.0468 (10)	−0.0107 (7)	−0.0121 (8)	0.0075 (7)
V3	0.0336 (12)	0.0215 (10)	0.0235 (11)	0.000	−0.0011 (9)	0.000
V4	0.0364 (12)	0.0185 (10)	0.0239 (11)	0.000	0.0033 (10)	0.000
Co1	0.0360 (7)	0.0185 (6)	0.0335 (7)	0.0002 (5)	−0.0013 (6)	−0.0028 (5)
O1	0.154 (9)	0.028 (4)	0.025 (4)	0.007 (5)	0.006 (5)	−0.006 (3)
O2	0.031 (5)	0.049 (6)	0.042 (6)	0.000	0.010 (4)	0.000
O3	0.032 (5)	0.034 (5)	0.030 (5)	0.000	−0.015 (4)	0.000
O4	0.063 (5)	0.026 (3)	0.039 (4)	0.003 (3)	0.008 (3)	0.006 (3)
O5	0.045 (6)	0.114 (10)	0.038 (6)	0.000	−0.013 (5)	0.000
O6	0.038 (6)	0.125 (11)	0.041 (6)	0.000	0.012 (5)	0.000
O7	0.061 (5)	0.036 (4)	0.078 (5)	0.027 (4)	−0.043 (4)	−0.023 (4)
O8	0.044 (4)	0.037 (4)	0.084 (6)	−0.017 (3)	0.006 (4)	−0.016 (4)
O9	0.063 (7)	0.041 (6)	0.022 (5)	0.000	0.010 (5)	0.000
O10	0.046 (5)	0.029 (5)	0.021 (4)	0.000	0.001 (4)	0.000
O11	0.043 (4)	0.040 (4)	0.071 (5)	−0.015 (3)	−0.025 (4)	0.028 (4)
O12	0.028 (3)	0.040 (4)	0.081 (5)	0.004 (3)	0.003 (4)	0.034 (4)
O13	0.035 (10)	0.017 (8)	0.019 (10)	0.003 (6)	0.005 (7)	−0.003 (6)
O13'	0.029 (9)	0.040 (11)	0.013 (9)	0.004 (7)	0.003 (6)	−0.013 (7)
O14	0.027 (7)	0.017 (8)	0.039 (9)	−0.003 (6)	−0.008 (7)	0.003 (6)
O14'	0.033 (8)	0.020 (9)	0.047 (11)	−0.001 (7)	−0.007 (7)	0.000 (7)
O15	0.020 (8)	0.030 (9)	0.041 (9)	0.007 (6)	0.009 (6)	0.004 (7)
O15'	0.028 (9)	0.016 (7)	0.030 (8)	−0.001 (6)	−0.003 (6)	−0.003 (6)
O16	0.036 (8)	0.014 (8)	0.040 (10)	0.006 (7)	−0.008 (7)	0.008 (6)
O16'	0.036 (8)	0.020 (8)	0.037 (9)	−0.002 (7)	0.006 (7)	−0.003 (6)
O17	0.046 (4)	0.027 (4)	0.081 (5)	−0.009 (3)	0.024 (4)	−0.029 (4)
O18	0.067 (5)	0.013 (3)	0.070 (5)	0.006 (3)	−0.035 (4)	−0.008 (3)
O19	0.033 (8)	0.018 (7)	0.024 (7)	0.001 (6)	−0.003 (6)	0.006 (6)
O19'	0.037 (8)	0.020 (7)	0.014 (7)	−0.003 (6)	0.008 (6)	−0.004 (5)
O20	0.057 (10)	0.010 (7)	0.020 (7)	0.005 (7)	−0.005 (7)	−0.006 (5)
O20'	0.040 (9)	0.028 (8)	0.045 (10)	0.002 (7)	0.014 (7)	0.009 (7)
O21	0.031 (9)	0.020 (8)	0.028 (9)	0.011 (6)	−0.005 (7)	−0.001 (6)
O21'	0.029 (9)	0.020 (8)	0.041 (11)	0.002 (6)	−0.011 (7)	0.006 (7)
O22	0.027 (10)	0.042 (10)	0.040 (11)	−0.003 (8)	0.017 (7)	−0.004 (8)
O22'	0.034 (10)	0.022 (8)	0.034 (10)	0.011 (8)	0.004 (8)	−0.001 (7)
O23	0.035 (7)	0.010 (5)	0.027 (6)	0.010 (5)	0.005 (5)	−0.001 (5)
O24	0.025 (6)	0.009 (5)	0.031 (6)	−0.003 (4)	−0.005 (5)	0.004 (5)
O25	0.025 (6)	0.021 (6)	0.030 (6)	−0.009 (5)	−0.004 (5)	−0.006 (5)
O26	0.031 (6)	0.026 (6)	0.017 (6)	0.002 (5)	0.005 (5)	0.007 (5)
O1W	0.049 (6)	0.033 (5)	0.051 (6)	0.000	0.000 (5)	0.000
O2W	0.145 (13)	0.26 (2)	0.146 (13)	0.044 (14)	0.035 (11)	−0.015 (14)
N1	0.044 (5)	0.025 (4)	0.045 (5)	−0.001 (4)	−0.002 (4)	0.004 (4)
N2	0.041 (4)	0.029 (4)	0.032 (4)	0.001 (4)	0.000 (4)	0.003 (3)
N3	0.068 (6)	0.033 (5)	0.045 (5)	0.005 (4)	0.003 (5)	−0.018 (4)
N4	0.046 (5)	0.024 (4)	0.049 (5)	0.004 (4)	−0.003 (4)	0.005 (4)
N5	0.044 (5)	0.020 (4)	0.053 (5)	−0.005 (4)	0.000 (4)	0.001 (4)
N6	0.038 (4)	0.028 (4)	0.045 (5)	−0.003 (4)	0.003 (4)	−0.004 (4)
C1	0.045 (6)	0.037 (5)	0.047 (6)	0.016 (5)	−0.003 (5)	−0.005 (5)
C2	0.036 (5)	0.038 (5)	0.036 (5)	0.000 (5)	0.003 (4)	0.012 (5)
C4	0.057 (7)	0.074 (8)	0.049 (7)	0.001 (7)	−0.020 (6)	−0.006 (6)
C5	0.047 (6)	0.033 (5)	0.062 (7)	−0.009 (5)	0.007 (5)	0.016 (5)
C6	0.033 (5)	0.044 (6)	0.045 (6)	−0.009 (5)	0.004 (5)	−0.010 (5)

### Geometric parameters (Å, °)

**Table:**

Si1—O23	1.623 (10)	V2—O14'	1.811 (16)
Si1—O25	1.628 (11)	V2—O20'	1.949 (16)
Si1—O24	1.655 (11)	V2—O16'	1.974 (15)
Si1—O26	1.674 (11)	V2—O16	2.002 (14)
Mo1—O1	1.665 (7)	V2—O20	2.022 (13)
Mo1—O14'	1.742 (16)	V2—O14	2.117 (16)
Mo1—O13'	1.793 (18)	V2—O22	2.156 (17)
Mo1—O13	1.839 (18)	V2—O24	2.441 (10)
Mo1—O14	1.871 (14)	V2—O25	2.454 (10)
Mo1—O12	2.046 (6)	V3—O9	1.614 (9)
Mo1—O11	2.067 (6)	V3—O11	1.918 (7)
Mo1—O24	2.365 (11)	V3—O12	1.941 (7)
Mo1—O23	2.409 (11)	V3—Mo1^i^	3.1080 (14)
Mo1—V3	3.1080 (14)	V4—O10	1.649 (8)
Mo2—O2	1.666 (9)	V4—O17	1.928 (7)
Mo2—O15'	1.686 (15)	V4—O18	1.939 (6)
Mo2—O15	1.980 (15)	V4—Mo4^i^	3.1144 (14)
Mo2—O11	2.046 (7)	Co1—N6	1.948 (8)
Mo2—O23	2.422 (11)	Co1—N1	1.952 (8)
Mo2—V3	3.081 (2)	Co1—N5	1.961 (8)
Mo3—O16'	1.643 (15)	Co1—N4	1.962 (8)
Mo3—O3	1.698 (8)	Co1—N2	1.967 (8)
Mo3—O16	1.952 (14)	Co1—N3	1.975 (8)
Mo3—O12	2.060 (7)	O25—O25^i^	1.79 (2)
Mo3—O24	2.400 (11)	N1—C1	1.488 (13)
Mo3—V3	3.114 (3)	N1—H1C	0.9000
Mo4—O19'	1.649 (12)	N1—H1D	0.9000
Mo4—O20'	1.671 (16)	N2—C2	1.494 (11)
Mo4—O4	1.689 (6)	N2—H2C	0.9000
Mo4—O20	1.923 (14)	N2—H2D	0.9000
Mo4—O19	1.994 (13)	N3—C3	1.41 (2)
Mo4—O18	2.032 (7)	N3—C3'	1.55 (3)
Mo4—O17	2.059 (7)	N3—H3C	0.9000
Mo4—O26	2.389 (11)	N3—H3D	0.9000
Mo4—O25	2.429 (11)	N4—C4	1.509 (13)
Mo4—V4	3.1144 (14)	N4—H4C	0.9000
Mo5—O5	1.669 (10)	N4—H4D	0.9000
Mo5—O21'	1.724 (17)	N5—C5	1.467 (13)
Mo5—O21	1.912 (15)	N5—H5C	0.9000
Mo5—O17	2.058 (7)	N5—H5D	0.9000
Mo5—O26	2.422 (11)	N6—C6	1.497 (12)
Mo5—V4	3.082 (2)	N6—H6C	0.9000
Mo6—O6	1.656 (10)	N6—H6D	0.9000
Mo6—O22'	1.777 (17)	C1—C2	1.521 (14)
Mo6—O22	1.835 (19)	C1—H1A	0.9700
Mo6—O18	2.047 (6)	C1—H1B	0.9700
Mo6—O25	2.355 (11)	C2—H2A	0.9700
Mo6—V4	3.100 (2)	C2—H2B	0.9700
V1—O7	1.579 (6)	C3—C4	1.67 (2)
V1—O13'	1.807 (18)	C3—H3A	0.9700
V1—O21'	1.844 (17)	C3—H3B	0.9700
V1—O15'	1.910 (15)	C3'—C4	1.30 (3)
V1—O19'	1.927 (12)	C3'—H3'1	0.9700
V1—O15	2.013 (15)	C3'—H3'2	0.9700
V1—O19	2.028 (12)	C4—H4A	0.9700
V1—O21	2.079 (16)	C4—H4B	0.9700
V1—O13	2.099 (17)	C5—C6	1.498 (14)
V1—O26	2.328 (11)	C5—H5A	0.9700
V1—O23	2.395 (11)	C5—H5B	0.9700
V2—O8	1.573 (7)	C6—H6A	0.9700
V2—O22'	1.794 (16)	C6—H6B	0.9700
			
O23^i^—Si1—O23	71.8 (8)	O13'—V1—O21	160.4 (6)
O23^i^—Si1—O25	177.4 (6)	O15'—V1—O21	88.4 (6)
O23—Si1—O25	110.7 (6)	O19'—V1—O21	92.7 (6)
O23^i^—Si1—O25^i^	110.7 (6)	O15—V1—O21	98.7 (7)
O23—Si1—O25^i^	177.4 (6)	O19—V1—O21	81.1 (6)
O25—Si1—O25^i^	66.7 (8)	O7—V1—O13	91.2 (5)
O23^i^—Si1—O24	109.4 (6)	O21'—V1—O13	157.3 (6)
O23—Si1—O24	69.5 (6)	O15'—V1—O13	89.7 (6)
O25—Si1—O24	71.8 (5)	O19'—V1—O13	86.8 (6)
O25^i^—Si1—O24	109.0 (6)	O15—V1—O13	80.5 (7)
O23^i^—Si1—O24^i^	69.5 (6)	O19—V1—O13	99.8 (6)
O23—Si1—O24^i^	109.4 (6)	O21—V1—O13	176.3 (6)
O25—Si1—O24^i^	109.0 (6)	O7—V1—O26	157.4 (4)
O25^i^—Si1—O24^i^	71.8 (5)	O13'—V1—O26	87.9 (6)
O24—Si1—O24^i^	71.0 (7)	O21'—V1—O26	57.3 (6)
O23^i^—Si1—O26^i^	68.0 (5)	O15'—V1—O26	87.9 (5)
O23—Si1—O26^i^	108.7 (6)	O19'—V1—O26	56.6 (5)
O25—Si1—O26^i^	110.9 (6)	O15—V1—O26	108.6 (5)
O25^i^—Si1—O26^i^	72.8 (6)	O19—V1—O26	73.7 (4)
O24—Si1—O26^i^	177.3 (6)	O21—V1—O26	72.6 (5)
O24^i^—Si1—O26^i^	108.1 (5)	O13—V1—O26	104.2 (5)
O23^i^—Si1—O26	108.7 (6)	O7—V1—O23	156.4 (4)
O23—Si1—O26	68.0 (5)	O13'—V1—O23	58.5 (6)
O25—Si1—O26	72.8 (6)	O21'—V1—O23	87.5 (5)
O25^i^—Si1—O26	110.9 (6)	O15'—V1—O23	58.9 (5)
O24—Si1—O26	108.1 (5)	O19'—V1—O23	84.5 (5)
O24^i^—Si1—O26	177.3 (6)	O15—V1—O23	73.7 (5)
O26^i^—Si1—O26	72.7 (8)	O19—V1—O23	108.8 (5)
O1—Mo1—O14'	113.8 (6)	O21—V1—O23	106.5 (5)
O1—Mo1—O13'	113.8 (6)	O13—V1—O23	69.8 (5)
O14'—Mo1—O13'	84.9 (7)	O26—V1—O23	45.9 (4)
O1—Mo1—O13	94.8 (5)	O8—V2—O22'	108.2 (6)
O14'—Mo1—O13	98.9 (7)	O8—V2—O14'	112.0 (6)
O1—Mo1—O14	90.2 (6)	O22'—V2—O14'	139.7 (7)
O13'—Mo1—O14	98.4 (7)	O8—V2—O20'	111.1 (5)
O13—Mo1—O14	106.1 (7)	O22'—V2—O20'	92.3 (7)
O1—Mo1—O12	95.3 (4)	O14'—V2—O20'	71.9 (7)
O14'—Mo1—O12	91.6 (5)	O8—V2—O16'	110.3 (5)
O13'—Mo1—O12	149.5 (6)	O22'—V2—O16'	76.7 (7)
O13—Mo1—O12	161.1 (6)	O14'—V2—O16'	90.9 (6)
O14—Mo1—O12	89.9 (5)	O20'—V2—O16'	138.5 (6)
O1—Mo1—O11	98.0 (4)	O8—V2—O16	91.9 (5)
O14'—Mo1—O11	146.5 (5)	O22'—V2—O16	93.9 (7)
O13'—Mo1—O11	91.8 (6)	O14'—V2—O16	86.6 (7)
O13—Mo1—O11	88.1 (6)	O20'—V2—O16	152.9 (6)
O14—Mo1—O11	163.0 (5)	O8—V2—O20	91.5 (5)
O12—Mo1—O11	74.7 (3)	O22'—V2—O20	85.3 (7)
O1—Mo1—O24	153.5 (4)	O14'—V2—O20	91.9 (7)
O14'—Mo1—O24	56.2 (6)	O16'—V2—O20	155.1 (6)
O13'—Mo1—O24	90.8 (5)	O16—V2—O20	176.6 (6)
O13—Mo1—O24	110.6 (5)	O8—V2—O14	92.8 (5)
O14—Mo1—O24	75.8 (5)	O22'—V2—O14	158.3 (7)
O12—Mo1—O24	62.8 (3)	O20'—V2—O14	84.9 (6)
O11—Mo1—O24	90.6 (3)	O16'—V2—O14	91.2 (6)
O1—Mo1—O23	157.1 (4)	O16—V2—O14	79.6 (6)
O14'—Mo1—O23	87.8 (6)	O20—V2—O14	99.9 (6)
O13'—Mo1—O23	58.3 (6)	O8—V2—O22	92.2 (5)
O13—Mo1—O23	73.5 (5)	O14'—V2—O22	154.8 (7)
O14—Mo1—O23	111.8 (5)	O20'—V2—O22	93.4 (7)
O12—Mo1—O23	91.3 (3)	O16'—V2—O22	87.0 (7)
O11—Mo1—O23	62.7 (3)	O16—V2—O22	100.1 (7)
O24—Mo1—O23	46.1 (4)	O20—V2—O22	80.1 (7)
O2—Mo2—O15'^i^	110.9 (6)	O14—V2—O22	175.0 (6)
O2—Mo2—O15'	110.9 (6)	O8—V2—O24	157.4 (4)
O15'^i^—Mo2—O15'	81.7 (10)	O22'—V2—O24	88.1 (6)
O2—Mo2—O15^i^	92.3 (5)	O14'—V2—O24	54.0 (5)
O15'—Mo2—O15^i^	97.6 (6)	O20'—V2—O24	82.9 (5)
O2—Mo2—O15	92.3 (5)	O16'—V2—O24	57.3 (5)
O15'^i^—Mo2—O15	97.6 (6)	O16—V2—O24	71.0 (5)
O15^i^—Mo2—O15	109.1 (10)	O20—V2—O24	105.7 (5)
O2—Mo2—O11	97.5 (3)	O14—V2—O24	70.1 (5)
O15'^i^—Mo2—O11	150.9 (6)	O22—V2—O24	105.0 (5)
O15'—Mo2—O11	94.4 (5)	O8—V2—O25	156.3 (4)
O15^i^—Mo2—O11	160.7 (5)	O22'—V2—O25	56.0 (6)
O15—Mo2—O11	87.2 (5)	O14'—V2—O25	85.1 (6)
O2—Mo2—O11^i^	97.5 (3)	O20'—V2—O25	57.2 (5)
O15'^i^—Mo2—O11^i^	94.4 (5)	O16'—V2—O25	84.6 (5)
O15'—Mo2—O11^i^	150.9 (6)	O16—V2—O25	105.8 (5)
O15^i^—Mo2—O11^i^	87.2 (5)	O20—V2—O25	71.0 (5)
O15—Mo2—O11^i^	160.7 (5)	O14—V2—O25	105.6 (5)
O11—Mo2—O11^i^	75.0 (4)	O22—V2—O25	69.6 (5)
O2—Mo2—O23^i^	155.6 (3)	O24—V2—O25	46.3 (4)
O15'^i^—Mo2—O23^i^	60.3 (6)	O9—V3—O11^i^	113.3 (4)
O15'—Mo2—O23^i^	91.0 (6)	O9—V3—O11	113.3 (4)
O15^i^—Mo2—O23^i^	73.6 (5)	O11^i^—V3—O11	81.0 (4)
O15—Mo2—O23^i^	111.0 (5)	O9—V3—O12^i^	114.2 (4)
O11—Mo2—O23^i^	91.1 (3)	O11^i^—V3—O12^i^	80.5 (3)
O11^i^—Mo2—O23^i^	62.7 (3)	O11—V3—O12^i^	132.5 (4)
O2—Mo2—O23	155.6 (3)	O9—V3—O12	114.2 (4)
O15'^i^—Mo2—O23	91.0 (6)	O11^i^—V3—O12	132.5 (4)
O15'—Mo2—O23	60.3 (6)	O11—V3—O12	80.5 (3)
O15^i^—Mo2—O23	111.0 (5)	O12^i^—V3—O12	80.6 (4)
O15—Mo2—O23	73.6 (5)	O10—V4—O17^i^	116.4 (3)
O11—Mo2—O23	62.7 (3)	O10—V4—O17	116.4 (3)
O11^i^—Mo2—O23	91.1 (3)	O17^i^—V4—O17	81.9 (4)
O23^i^—Mo2—O23	46.3 (5)	O10—V4—O18	111.9 (3)
O16'^i^—Mo3—O16'	77.6 (10)	O17^i^—V4—O18	131.7 (4)
O16'^i^—Mo3—O3	114.1 (6)	O17—V4—O18	79.6 (3)
O16'—Mo3—O3	114.1 (6)	O10—V4—O18^i^	111.9 (3)
O16'^i^—Mo3—O16	96.3 (7)	O17^i^—V4—O18^i^	79.6 (3)
O3—Mo3—O16	95.1 (5)	O17—V4—O18^i^	131.7 (4)
O16'—Mo3—O16^i^	96.3 (7)	O18—V4—O18^i^	80.4 (4)
O3—Mo3—O16^i^	95.1 (5)	N6—Co1—N1	94.2 (3)
O16—Mo3—O16^i^	111.1 (9)	N6—Co1—N5	83.9 (3)
O16'^i^—Mo3—O12	151.0 (6)	N1—Co1—N5	90.3 (3)
O16'—Mo3—O12	96.3 (5)	N6—Co1—N4	91.0 (3)
O3—Mo3—O12	94.4 (3)	N1—Co1—N4	174.7 (4)
O16—Mo3—O12	86.1 (5)	N5—Co1—N4	91.3 (3)
O16^i^—Mo3—O12	159.5 (5)	N6—Co1—N2	89.2 (3)
O16'^i^—Mo3—O12^i^	96.3 (5)	N1—Co1—N2	85.2 (3)
O16'—Mo3—O12^i^	151.0 (6)	N5—Co1—N2	171.5 (3)
O3—Mo3—O12^i^	94.4 (3)	N4—Co1—N2	93.9 (3)
O16—Mo3—O12^i^	159.5 (5)	N6—Co1—N3	175.3 (4)
O16^i^—Mo3—O12^i^	86.1 (5)	N1—Co1—N3	89.5 (4)
O12—Mo3—O12^i^	75.1 (4)	N5—Co1—N3	93.2 (4)
O16'^i^—Mo3—O24^i^	61.1 (6)	N4—Co1—N3	85.4 (4)
O16'—Mo3—O24^i^	91.1 (6)	N2—Co1—N3	94.0 (4)
O3—Mo3—O24^i^	153.4 (3)	V3—O11—Mo2	102.0 (3)
O16—Mo3—O24^i^	111.3 (5)	V3—O11—Mo1	102.5 (3)
O16^i^—Mo3—O24^i^	72.7 (5)	Mo2—O11—Mo1	130.1 (4)
O12—Mo3—O24^i^	91.0 (3)	V3—O12—Mo1	102.4 (3)
O12^i^—Mo3—O24^i^	62.0 (3)	V3—O12—Mo3	102.2 (3)
O16'^i^—Mo3—O24	91.1 (6)	Mo1—O12—Mo3	129.3 (4)
O16'—Mo3—O24	61.1 (6)	Mo1—O13—V1	123.2 (7)
O3—Mo3—O24	153.4 (3)	Mo1—O13'—V1	148.7 (12)
O16—Mo3—O24	72.7 (5)	Mo1—O14—V2	120.6 (7)
O16^i^—Mo3—O24	111.3 (5)	Mo1—O14'—V2	154.6 (9)
O12—Mo3—O24	62.0 (3)	Mo2—O15—V1	119.4 (7)
O12^i^—Mo3—O24	91.0 (3)	Mo2—O15'—V1	146.9 (9)
O24^i^—Mo3—O24	47.2 (5)	Mo3—O16—V2	122.4 (8)
O19'—Mo4—O20'	76.7 (7)	Mo3—O16'—V2	146.5 (9)
O19'—Mo4—O4	113.8 (5)	V4—O17—Mo5	101.2 (3)
O20'—Mo4—O4	112.9 (6)	V4—O17—Mo4	102.7 (3)
O19'—Mo4—O20	98.2 (6)	Mo5—O17—Mo4	129.3 (4)
O4—Mo4—O20	91.8 (5)	V4—O18—Mo4	103.3 (3)
O20'—Mo4—O19	96.1 (6)	V4—O18—Mo6	102.1 (3)
O4—Mo4—O19	92.1 (4)	Mo4—O18—Mo6	130.9 (4)
O20—Mo4—O19	112.2 (5)	Mo4—O19—V1	117.5 (6)
O19'—Mo4—O18	148.7 (5)	Mo4—O19'—V1	148.0 (7)
O20'—Mo4—O18	96.9 (5)	Mo4—O20—V2	122.8 (7)
O4—Mo4—O18	97.0 (3)	Mo4—O20'—V2	146.1 (9)
O20—Mo4—O18	85.6 (4)	Mo5—O21—V1	120.0 (7)
O19—Mo4—O18	159.7 (4)	Mo5—O21'—V1	151.3 (10)
O19'—Mo4—O17	95.5 (5)	Mo6—O22—V2	120.3 (8)
O20'—Mo4—O17	149.7 (6)	Mo6—O22'—V2	152.0 (10)
O4—Mo4—O17	97.1 (3)	Si1—O23—V1	122.4 (6)
O20—Mo4—O17	159.0 (4)	Si1—O23—Mo1	121.6 (6)
O19—Mo4—O17	86.5 (4)	V1—O23—Mo1	92.3 (3)
O18—Mo4—O17	74.5 (3)	Si1—O23—Mo2	120.8 (5)
O19'—Mo4—O26	57.5 (5)	V1—O23—Mo2	91.4 (4)
O20'—Mo4—O26	88.2 (6)	Mo1—O23—Mo2	101.1 (4)
O4—Mo4—O26	155.6 (4)	Si1—O24—Mo1	122.4 (6)
O20—Mo4—O26	111.5 (5)	Si1—O24—Mo3	120.6 (5)
O19—Mo4—O26	72.9 (4)	Mo1—O24—Mo3	102.3 (4)
O18—Mo4—O26	92.1 (3)	Si1—O24—V2	120.6 (6)
O17—Mo4—O26	63.7 (3)	Mo1—O24—V2	92.3 (3)
O19'—Mo4—O25	90.1 (6)	Mo3—O24—V2	91.4 (4)
O20'—Mo4—O25	60.0 (6)	Si1—O25—Mo6	124.2 (6)
O4—Mo4—O25	153.6 (4)	Si1—O25—Mo4	119.4 (6)
O20—Mo4—O25	73.1 (5)	Mo6—O25—Mo4	101.7 (4)
O19—Mo4—O25	113.5 (5)	Si1—O25—V2	121.3 (6)
O18—Mo4—O25	61.1 (3)	Mo6—O25—V2	92.2 (4)
O17—Mo4—O25	91.2 (3)	Mo4—O25—V2	90.4 (4)
O26—Mo4—O25	48.0 (4)	Si1—O26—V1	123.7 (6)
O5—Mo5—O21'	114.2 (6)	Si1—O26—Mo4	119.4 (6)
O5—Mo5—O21'^i^	114.2 (6)	V1—O26—Mo4	93.6 (4)
O21'—Mo5—O21'^i^	84.6 (11)	Si1—O26—Mo5	119.5 (6)
O5—Mo5—O21^i^	93.6 (5)	V1—O26—Mo5	93.4 (4)
O21'—Mo5—O21^i^	96.8 (6)	Mo4—O26—Mo5	101.3 (4)
O21'^i^—Mo5—O21^i^	20.9 (5)	C1—N1—Co1	110.8 (6)
O5—Mo5—O21	93.6 (5)	C1—N1—H1C	109.5
O21'^i^—Mo5—O21	96.8 (6)	Co1—N1—H1C	109.5
O21^i^—Mo5—O21	102.9 (10)	C1—N1—H1D	109.5
O5—Mo5—O17	95.9 (4)	Co1—N1—H1D	109.5
O21'—Mo5—O17	91.5 (6)	H1C—N1—H1D	108.1
O21'^i^—Mo5—O17	148.6 (6)	C2—N2—Co1	110.3 (5)
O21^i^—Mo5—O17	163.5 (5)	C2—N2—H2C	109.6
O21—Mo5—O17	90.0 (5)	Co1—N2—H2C	109.6
O5—Mo5—O17^i^	95.9 (4)	C2—N2—H2D	109.6
O21'—Mo5—O17^i^	148.6 (6)	Co1—N2—H2D	109.6
O21'^i^—Mo5—O17^i^	91.5 (6)	H2C—N2—H2D	108.1
O21^i^—Mo5—O17^i^	90.0 (5)	C3—N3—Co1	114.3 (10)
O21—Mo5—O17^i^	163.5 (5)	C3'—N3—Co1	109.4 (12)
O17—Mo5—O17^i^	75.7 (4)	C3—N3—H3C	108.7
O5—Mo5—O26	154.3 (3)	C3'—N3—H3C	131.7
O21'—Mo5—O26	56.1 (6)	Co1—N3—H3C	108.7
O21'^i^—Mo5—O26	89.6 (6)	C3—N3—H3D	108.7
O21^i^—Mo5—O26	110.5 (5)	C3'—N3—H3D	86.9
O21—Mo5—O26	73.2 (5)	Co1—N3—H3D	108.7
O17—Mo5—O26	63.0 (3)	H3C—N3—H3D	107.6
O17^i^—Mo5—O26	92.8 (3)	C4—N4—Co1	109.1 (7)
O5—Mo5—O26^i^	154.3 (3)	C4—N4—H4C	109.9
O21'—Mo5—O26^i^	89.6 (6)	Co1—N4—H4C	109.9
O21'^i^—Mo5—O26^i^	56.1 (6)	C4—N4—H4D	109.9
O21^i^—Mo5—O26^i^	73.2 (5)	Co1—N4—H4D	109.9
O21—Mo5—O26^i^	110.5 (5)	H4C—N4—H4D	108.3
O17—Mo5—O26^i^	92.8 (3)	C5—N5—Co1	109.2 (6)
O17^i^—Mo5—O26^i^	63.0 (3)	C5—N5—H5C	109.8
O26—Mo5—O26^i^	48.3 (5)	Co1—N5—H5C	109.8
O6—Mo6—O22'	113.0 (6)	C5—N5—H5D	109.8
O6—Mo6—O22'^i^	113.0 (6)	Co1—N5—H5D	109.8
O22'—Mo6—O22'^i^	87.5 (11)	H5C—N5—H5D	108.3
O6—Mo6—O22	91.5 (5)	C6—N6—Co1	112.7 (6)
O22'^i^—Mo6—O22	98.6 (6)	C6—N6—H6C	109.1
O6—Mo6—O22^i^	91.5 (5)	Co1—N6—H6C	109.1
O22'—Mo6—O22^i^	98.6 (6)	C6—N6—H6D	109.1
O22—Mo6—O22^i^	102.4 (13)	Co1—N6—H6D	109.1
O6—Mo6—O18^i^	96.9 (4)	H6C—N6—H6D	107.8
O22'—Mo6—O18^i^	148.3 (6)	N1—C1—C2	105.7 (8)
O22'^i^—Mo6—O18^i^	90.3 (6)	N1—C1—H1A	110.6
O22—Mo6—O18^i^	164.3 (6)	C2—C1—H1A	110.6
O22^i^—Mo6—O18^i^	90.6 (7)	N1—C1—H1B	110.6
O6—Mo6—O18	96.9 (4)	C2—C1—H1B	110.6
O22'—Mo6—O18	90.3 (6)	H1A—C1—H1B	108.7
O22'^i^—Mo6—O18	148.3 (6)	N2—C2—C1	108.3 (8)
O22—Mo6—O18	90.6 (7)	N2—C2—H2A	110.0
O22^i^—Mo6—O18	164.3 (6)	C1—C2—H2A	110.0
O18^i^—Mo6—O18	75.4 (3)	N2—C2—H2B	110.0
O6—Mo6—O25^i^	155.7 (3)	C1—C2—H2B	110.0
O22'—Mo6—O25^i^	90.1 (6)	H2A—C2—H2B	108.4
O22'^i^—Mo6—O25^i^	58.4 (6)	N3—C3—C4	102.5 (14)
O22—Mo6—O25^i^	111.7 (5)	N3—C3—H3A	111.3
O22^i^—Mo6—O25^i^	77.2 (6)	C4—C3—H3A	111.3
O18^i^—Mo6—O25^i^	62.3 (4)	N3—C3—H3B	111.3
O18—Mo6—O25^i^	90.0 (3)	C4—C3—H3B	111.3
O6—Mo6—O25	155.7 (3)	H3A—C3—H3B	109.2
O22'—Mo6—O25	58.4 (6)	C4—C3'—N3	115 (2)
O22'^i^—Mo6—O25	90.1 (6)	C4—C3'—H3'1	108.6
O22—Mo6—O25	77.2 (6)	N3—C3'—H3'1	108.6
O22^i^—Mo6—O25	111.7 (5)	C4—C3'—H3'2	108.6
O18^i^—Mo6—O25	90.0 (3)	N3—C3'—H3'2	108.6
O18—Mo6—O25	62.3 (4)	H3'1—C3'—H3'2	107.5
O25^i^—Mo6—O25	44.7 (5)	C3'—C4—N4	118.0 (16)
O7—V1—O13'	105.6 (6)	N4—C4—C3	105.3 (10)
O7—V1—O21'	110.5 (5)	C3'—C4—H4A	118.0
O13'—V1—O21'	143.8 (7)	N4—C4—H4A	110.7
O7—V1—O15'	109.0 (6)	C3—C4—H4A	110.7
O13'—V1—O15'	93.0 (7)	C3'—C4—H4B	87.9
O21'—V1—O15'	77.6 (7)	N4—C4—H4B	110.7
O7—V1—O19'	109.0 (5)	C3—C4—H4B	110.7
O13'—V1—O19'	74.2 (7)	H4A—C4—H4B	108.8
O21'—V1—O19'	91.8 (6)	N5—C5—C6	107.6 (8)
O15'—V1—O19'	141.9 (6)	N5—C5—H5A	110.2
O7—V1—O15	89.9 (5)	C6—C5—H5A	110.2
O13'—V1—O15	89.3 (7)	N5—C5—H5B	110.2
O21'—V1—O15	92.9 (7)	C6—C5—H5B	110.2
O19'—V1—O15	157.5 (6)	H5A—C5—H5B	108.5
O7—V1—O19	87.6 (5)	N6—C6—C5	105.8 (8)
O13'—V1—O19	91.6 (7)	N6—C6—H6A	110.6
O21'—V1—O19	87.7 (6)	C5—C6—H6A	110.6
O15'—V1—O19	160.8 (6)	N6—C6—H6B	110.6
O15—V1—O19	177.5 (6)	C5—C6—H6B	110.6
O7—V1—O21	92.4 (5)	H6A—C6—H6B	108.7
			
Co1—N1—C1—C2	40.8 (9)	N3—C3'—C4—C3	71 (4)
Co1—N2—C2—C1	33.3 (9)	Co1—N4—C4—C3'	−19 (2)
N1—C1—C2—N2	−47.4 (10)	Co1—N4—C4—C3	−39.7 (12)
C3'—N3—C3—C4	45 (3)	N3—C3—C4—C3'	−76 (4)
Co1—N3—C3—C4	−40.2 (16)	N3—C3—C4—N4	51.1 (16)
C3—N3—C3'—C4	−102 (4)	Co1—N5—C5—C6	44.7 (10)
Co1—N3—C3'—C4	4(3)	Co1—N6—C6—C5	30.4 (10)
N3—C3'—C4—N4	10 (3)	N5—C5—C6—N6	−47.8 (11)

Symmetry codes: (i) *x*, −*y*+3/2, *z*.

### Hydrogen-bond geometry (Å, °)

**Table:**

*D*—H···*A*	*D*—H	H···*A*	*D*···*A*	*D*—H···*A*
N1—H1C···O4^ii^	0.90	2.08	2.918 (10)	154
N1—H1D···O14^iii^	0.90	2.48	2.988 (16)	117
N2—H2D···O10^iv^	0.90	2.24	3.040 (9)	148
N2—H2C···O12^v^	0.90	2.22	3.034 (11)	151
N3—H3D···O19^iii^	0.90	1.97	2.765 (15)	147
N4—H4D···O3^vi^	0.90	2.17	3.042 (9)	164
N4—H4C···O15^vii^	0.90	2.09	2.770 (16)	131
N5—H5D···O7^iii^	0.90	2.26	2.923 (10)	130
N5—H5C···O7^vii^	0.90	2.12	2.905 (11)	145
N5—H5D···O13^iii^	0.90	2.07	2.835 (18)	142
N6—H6C···O4^ii^	0.90	2.31	3.126 (11)	150
N6—H6D···O10^iv^	0.90	2.57	3.109 (9)	120
N6—H6C···O18^ii^	0.90	2.37	2.972 (11)	124

Symmetry codes: (ii) *x*−1/2, *y*, −*z*+3/2; (iii) −*x*+1, −*y*+1, −*z*+1; (iv) *x*−1/2, −*y*+3/2, −*z*+3/2; (v) *x*−1/2, *y*, −*z*+1/2; (vi) *x*−1/2, −*y*+3/2, −*z*+1/2; (vii) *x*−1, *y*, *z*.
